# A 3D anatomical atlas of appendage musculature in the chelicerate arthropod *Limulus polyphemus*

**DOI:** 10.1371/journal.pone.0191400

**Published:** 2018-02-14

**Authors:** Russell D. C. Bicknell, Ada J. Klinkhamer, Richard J. Flavel, Stephen Wroe, John R. Paterson

**Affiliations:** 1 Palaeoscience Research Centre, School of Environmental and Rural Science, University of New England, Armidale, Australia; 2 FEARLab, Palaeoscience Research Centre, School of Environmental and Rural Science, University of New England, Armidale, Australia; 3 Agronomy and Soil Science, School of Environmental and Rural Science, University of New England, Armidale, Australia; INRA, FRANCE

## Abstract

*Limulus polyphemus*, an archetypal chelicerate taxon, has interested both biological and paleontological researchers due to its unique suite of anatomical features and as a useful modern analogue for fossil arthropod groups. To assist the study and documentation of this iconic taxon, we present a 3D atlas on the appendage musculature, with specific focus on the muscles of the cephalothoracic appendages. As *L*. *polyphemus* appendage musculature has been the focus of extensive study, depicting the muscles in 3D will facilitate a more complete understanding thereof for future researchers. A large museum specimen was CT scanned to illustrate the major exoskeletal features of *L*. *polyphemus*. Micro-CT scans of iodine-stained appendages from fresh, non-museum specimens were digitally dissected to interactively depict appendage sections and muscles. This study has revealed the presence of two new muscles: one within the pushing leg, located dorsally relative to all other patella muscles, and the other within the male pedipalp, located in the modified tibiotarsus. This atlas increases accessibility to important internal and external morphological features of *L*. *polyphemus* and reduces the need for destructive fresh tissue dissection of specimens. Scanning, digitally dissecting, and documenting taxa in 3D is a pivotal step towards creating permanent digital records of life on Earth.

## Introduction

Advances in modern computed tomography (CT or serial x-ray), micro-computed tomography (micro-CT), synchrotron-radiation micro-CT and magnetic resonance imaging (MRI) technology have presented researchers interested in biological form and function the opportunity to study complex internal and external anatomical morphological features, without destroying specimens [1–3]. This technology was pivotal in driving the modern ‘taxonomic renaissance’ and the understanding of external morphology and *in situ* internal features [1, 4]. The scans produced by these tools can be employed in 3D digital dissection and reconstruction of organisms in the form of interactive 3D PDFs [5, 6]. As anatomy is a three dimensional construct, 3D PDFs represent reality more accurately than 2D images in publications [7, 8]. Using interactive 3D models to illustrate organisms is a major step towards accurately documenting taxa and provides a platform for the easy and rapid dissemination of information [9].

The majority of research depicting organisms in 3D has considered vertebrates, while invertebrates were largely excluded from digital documentation until recently [7]. Invertebrate groups that have been documented in 3D include chelicerates [10–12], crabs [13], earth worms [2], gastropods [14–18] (to name a few), insects [19–23], monoplacophorans [24], polychaetes [25] and sea urchins [4]. Although not comprehensive, this list illustrates how few invertebrate groups have been digitally documented, compared to the number of known invertebrate clades. To expand this list, one of the archetypal chelicerates, *Limulus polyphemus* (Linnaeus), was selected for CT scanning, digital dissection and interactive three-dimensional PDF construction. The similarity of *L*. *polyphemus* to some extinct xiphosurans (such as the Jurassic *Mesolimulus walchi* (Desmarest)), coupled with the size and lifestyle of *L*. *polyphemus*, has made the taxon one of the most extensively studied arthropods from both biological and paleontological perspectives [26]. An extensive record of *L*. *polyphemus* muscles will allow studies that employ *L*. *polyphemus* as a modern analogue to present possible muscle combinations and place functional limits on motion and feeding ability for extinct taxa. Even though the internal and external features of *L*. *polyphemus* are regularly depicted as 2D illustrations in various publications, *L*. *polyphemus* has never been documented in 3D [27]. This study presents a three-dimensional interactive model of *L*. *polyphemus* appendages and muscles, along with a written description of the studied musculature.

## Methods

Digital dissection was performed on one large (56-cm-long, including telson), dried, articulated female carcass of *Limulus polyphemus* (specimen number Va. 06) housed in the Natural History Museum of the University of New England (Armidale, New South Wales, Australia). It was scanned on a Siemens SOMATOM definition medical CT scanner at Armidale Radiology (Armidale, NSW, Australia). Scan data include 822 slices at a slice thickness of 0.75 mm. The scan was imported into Mimics 19.0 (Mimics 19.0, Materialise, Leuven, Belgium). A 3D model of the exoskeleton was created by segmenting the scan using the Mimics ‘thresholding tool’. The main exoskeletal components of interest were modelled separately for closer inspection and description.

In addition to the complete specimen, fresh appendages of *Limulus polyphemus* were obtained from the Marine Biological Laboratory of Woods Hole, USA to document the appendages and associated muscles in greater detail. These specimens include articulated examples of one chelicera, one male pedipalp, one walking leg, one pushing leg, one set of chilaria, one genital operculum, and one gill operculum. Appendages were submerged in a solution of 1% iodine metal dissolved in pure ethanol for 13 days, following the procedure outlined in [28] and the staining time suggested in [29]. The walking leg was re-stained for another 13 days, as the first staining process failed to sufficiently highlight the muscles. After staining, specimens were washed thoroughly in ethanol and placed in ethanol for 2 days to leach any additional iodine before being scanned in the micro-CT (GE-Phoenix V|tome|xs micro CT scanner with 240kV ‘Direct’ tube) at the University of New England (UNE). Iodine staining of the appendages prior to CT scanning facilitates the observation of detailed muscle structures, without damaging the specimen (see [25, 28, 29]). Furthermore, as iodine staining of arthropod specimens has previously identified microscopic features (e.g., single muscle fibers) [28], it is likely that all of the larger muscles have been discerned using this method. Micro-CT data of samples were captured using Datos acquisition software version 2.2.1 and reconstruction software version 2.2.1 RTM. The samples were mounted on the rotating stage and imaged using the previously determined optimal X-ray tube settings (150 kV, 200 μA, 200 ms integration time per projection, focal spot 4 μm diameter for the divergent, polychromatic source). Projections (3600 in 360 degrees) were captured using a 2000 x 1000 pixel ‘virtual’ (moving) detector array. The isotropic voxel side length varied with the sample size: 70.2 μm for the gill operculum, male appendage, walking and pushing legs, and set of chilaria and chelicera; 46.8 μm for the genital operculum. All scans were captured using the GE constant rotation CT function to improve acquisition time and sample movement during the scan.

The tomographs were imported into Mimics 19.0 to conduct a digital dissection, involving the segmentation of the exoskeleton and individual muscles in each appendage. Muscle descriptions were derived by studying the scans and 3D reconstructions, and identified using publications that previously detailed the muscles of interest [27, 30–35]. The muscles are numbered using Shultz’s numbering system [27]—which was an expansion of Lankester’s terminology [31]—to facilitate easy comparison between the reconstructions presented here and in Shultz’s monumental work. However, in some cases, the muscles were previously ascribed names and so these have also been included in the descriptions. A total of 32 individual muscles were identified and described. Once the digital dissection was complete and muscles identified, 3D models of the exoskeleton and individual muscles were exported from Mimics and 3D PDFs were generated using 3D Reviewer [36]. 3D PDFs were created for each appendage as well as the overall specimen. Distinct muscles and exoskeletal elements were assigned different colours so they could be more easily differentiated from each other. For the 3D PDFs of the walking leg, pushing leg and male pedipalp, the exoskeletal components and muscles that are common across the PDFs are coloured the same. The same approach was applied to the genital operculum and gill operculum PDFs. As there are muscles common to the walking leg, pushing leg and male pedipalp, the most anatomically informative muscle reconstructions from these three PDFs were used to construct the in-text figures. The 3D PDFs are supplementary files (S1–S8 Figs) that are available from the Dryad Digital Repository at the following DOI: 10.5061/dryad.vs044.

## Results

The results presented here provide a 3D atlas of the major external (exoskeletal) features of *L*. *polyphemus*, coupled with detailed images and descriptions of appendage musculature. During the construction of the atlas, two new muscles were identified: one from the male pedipalp and one within the pushing leg. Within the pushing leg, subdivisions for two muscles is presented. Interestingly, due to the additional (prolonged) staining of the walking leg, the muscles within this scan are shrunk in comparison to the pushing leg and male pedipalp. The interactive 3D models (S1–S8 Figs) should be considered in conjunction with the descriptions of the exoskeleton and muscles.

### Dorsal shield

**Fig 1:**
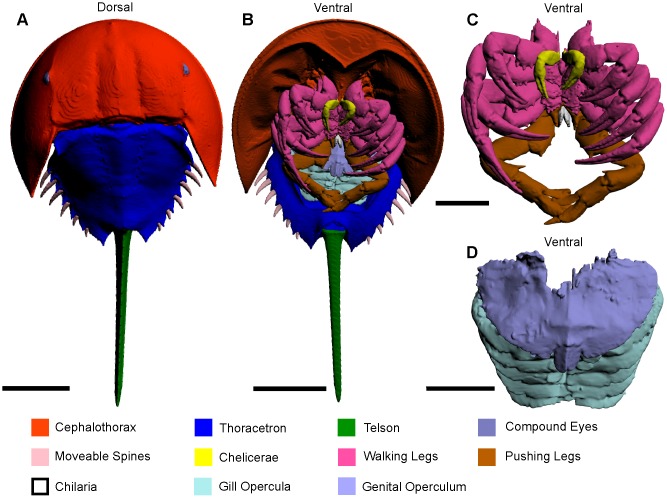
3D reconstruction of the complete museum specimen (va.06) of *Limulus polyphemus* in dorsal and ventral aspect, as modelled from CT scanning. (A) Dorsal view of entire exoskeleton. (B) Ventral view of entire exoskeleton. (C) Detail of cephalothoracic appendages. (D) Detail of thoracetronic appendages. The interactive 3D version of this file is available in the Supporting Information (S1 Fig). Scale bars 100 mm for A, B; 50 mm for C, D.

The dorsal shield of *Limulus polyphemus* consists of two major tagmata: the anterior cephalothorax, and the posterior thoracetron, both of which cover the more delicate ventral structures (Fig 1A and 1B) [26, 37–40]. The cephalothorax and thoracetron are connected by a soft arthrodial membrane hinge [38].

### Cephalothorax

The anterior section is semicircular, margined by a lipped flange, with two lateral compound eyes and central ocelli (Fig 1A and 1B) [38, 41]. Three ridges are present on the cephalothorax: the median ridge that runs from the membranous hinge to the ocelli and bisects the dorsal shield, and two lateral ophthalmic ridges that are slightly convergent anteriorly and intercept the adaxial sides of the compound eyes [42]. Between each ophthalmic ridge and the median ridge is a longitudinal furrow [43]. On the underside of the cephalothorax are six pairs of prosomal appendages and one set of opisthosomal appendages [40].

### Thoracetron

The hexagonal posterior section of the dorsal shield is bisected by a median ridge: a posterior continuation of the cephalothoracic median ridge (Fig 1A and 1B) [40, 42]. Flanking the median ridge are six pairs of entapophyseal pits: attachment sites for the six other opisthosomal appendages that are located on the underside of the thoracetron [40, 42]. Along the posterolateral margins of the thoracetron are six moveable spines housed within notches [40].

### Telson

The most posterior component of the *Limulus polyphemus* exoskeleton is the telson. This triangular ridged spine attaches to the opisthosoma with a ball joint (Fig 1A and 1B). The ball joint attachment allows the telson to be highly moveable and permits righting of an overturned individual [26, 38, 44]. The telson is capable of rotating through a complete circle and has full 180° range of motion in the vertical plane [45, 46].

### Appendages

*Limulus polyphemus* has 13 pairs of appendages (Fig 1B–1D). The first seven pairs of appendages are attached to the ventral side of the cephalothorax and are used for locomotion and feeding. The first six are segmented and the seventh is not. In order, starting from the anteriormost pair, there are the chelicerae (pair 1), walking legs (pairs 2–5), pushing legs (pair 6), and chilaria (pair 7). In males, the first pair of walking legs is modified during ontogeny into the so-called male pedipalp. There are six appendages attached to the ventral side of the thoracetron [39, 47]. The thoracetronic appendages are divided into two groups: the genital operculum (pair 8) and the five gill opercula that are used for respiration and swimming (pairs 9–13) [26, 39, 47].

### Chelicerae

**Fig 2:**
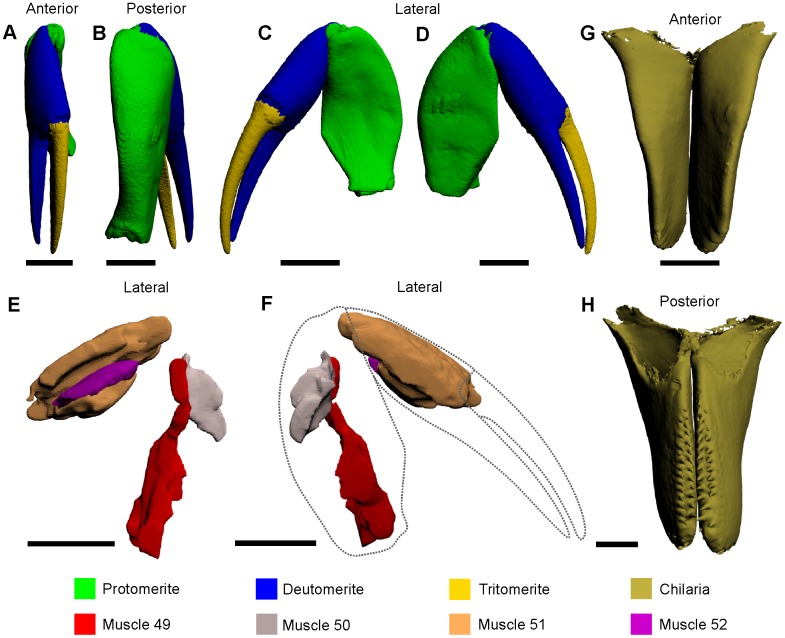
3D reconstruction of a single chelicera (A–D) and muscles therein (E, F) and the chilaria (G, H), as modelled from iodine staining and micro-CT–scanning. The 3D versions of these files are available in the Supporting Information (S2 and S6 Figs). Dotted lines in F outline the protomerite, deutomerite and tritomerite to indicate the relative positions of the muscles. All scale bars 5 mm.

The chelicerae are a pair of forward facing appendages and consist of three segments: the protomerite, deutomerite and tritomerite (Fig 2A–2D) [31, 47]. The tritomerite is a moveable arm that functions with the fixed deutomerite to clasp, grip and manipulate food to the mouth after mastication by the waking and pushing legs [26, 27, 31, 39, 48]. There are four muscles within the protomerite, deutomerite and tritomerite [27].

Muscle 49—This muscle has an origin on the dorsolateral and ventroproximal section of the protomerite and an insertion on the dorsal margin of the deutomerite (Fig 2E and 2F) [27]. This elongate muscle is located ventrally relative to muscle 50.
Muscle 50—This muscle has an origin on the dorsomedial and ventroproximal section of the protomerite and an insertion on the ventral margin of the deutomerite (Fig 2E and 2F) [27]. This muscle is located dorsally relative to muscle 49.
Muscle 51—This muscle has three origins on the dorsal section of the deutomerite and an insertion on the medial margin of the tritomerite (Fig 2E and 2F) [27]. This muscle has three major components that are all elongate and located posteriorly relative to muscle 52.
Muscle 52—This muscle has an origin on the lateral section of the deutomerite and an insertion on the lateral margin of the tritomerite (Fig 2E) [27]. This muscle is very thin, elongate and located anteriorly relative to muscle 51.

### Walking leg

**Fig 3:**
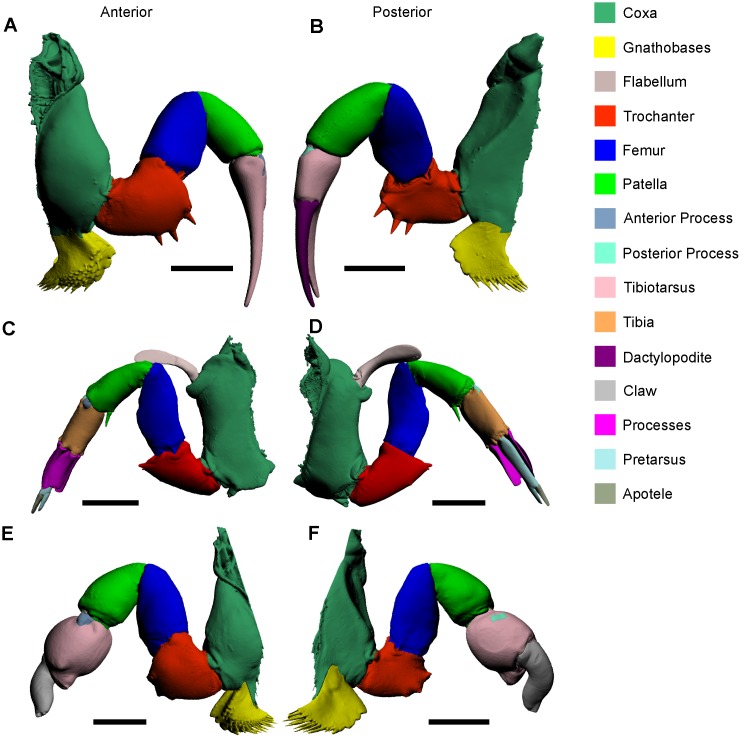
3D reconstructions of the walking leg (A, B), pushing leg (C, D), and male pedipalp (E, F) as modelled from micro-CT scanning. Reconstructions in the left column (A, C, E) are all anterior views and reconstructions in the right column (B, D, F) are all posterior views. These reconstructions should be studied in conjunction with the muscle reconstructions in Fig 4. The 3D versions of these files are available in the Supporting Information (S3, S4 and S5 Figs). Scale bars 15 mm for A, B, E, F; 20 mm for C, D.

**Fig 4:**
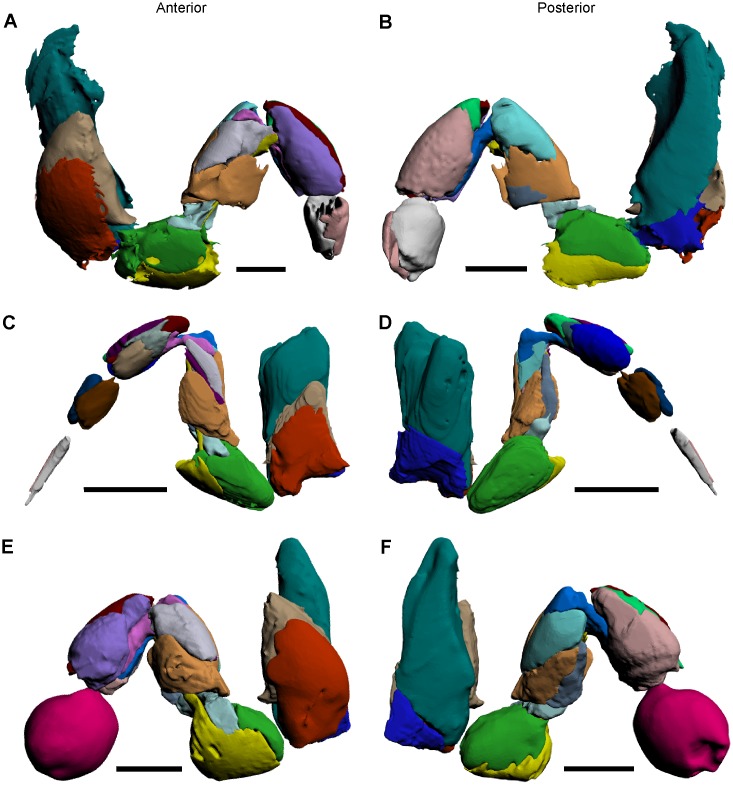
3D reconstructions of identified muscles within the walking leg (A, B), pushing leg (C, D), and male pedipalp (E, F), as modelled from iodine staining and micro-CT–scanning. A key is not included, as deeper muscles cannot easily be visualized in this figure. Individual muscles of specific prosomal appendages are illustrated separately in Figs 5–8. Reconstructions in the left column (A, C, E) are all anterior views and reconstructions in the right column (B, D, F) are all posterior views. These reconstructions should be considered in conjunction with the cephalothoracic appendage reconstructions in Fig 3. The 3D versions of these files are available in the Supporting Information (S3, S4 and S5 Figs). Scale bars 10 mm for A, B, E, F; 20 mm for C, D.

There are four pairs of chelate walking legs [47] in the set of female appendages, and three pairs in the set of male appendages, as the first walking leg is modified into the male pedipalp during ontogeny (Fig 3A and 3B) [49]. Walking legs have six segments (proximally to distally): the coxal endite that exhibits gnathobases; trochanter; patella; tibia; tibiotarsus; and the dactylopodite (referred to elsewhere as the claw or apotele) [26, 27, 31–34, 39, 49]. The dactylopodite is a moveable segment attached to the tibiotarsus, allowing these two segments to function together as claspers to move food to the gnathobases for mastication and shell breaking [49]. The main use of the walking legs is in locomotion and feeding, as they are radially arranged about the mouth [26, 39, 49–51]. There are 20 muscles in the walking legs (Fig 4A and 4B). Note that muscles 67–84 in the walking leg are the same as the male pedipalp, and muscles 67–82 in the walking leg are common to the pushing leg. The descriptions of the walking leg muscles 67–84 apply the male pedipalp, and the descriptions of walking leg muscles 67–82 apply to the pushing leg, so will not be redescribed in the pushing leg and male pedipalp sections.

**Fig 5:**
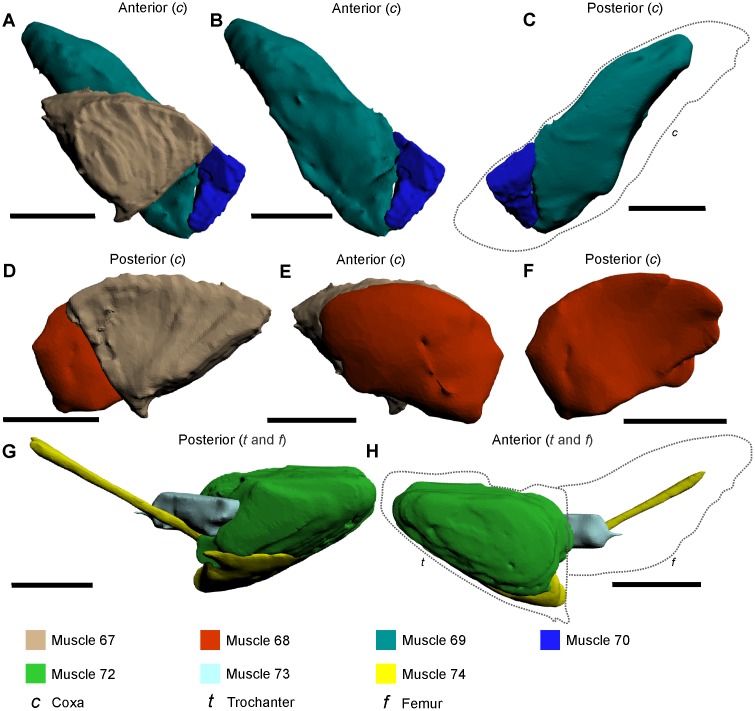
The muscles within the coxa, trochanter and femur of the walking legs, male pedipalp and pushing leg as modelled from iodine staining and micro-CT–scanning. The illustrated muscle reconstructions for A–F were selected from the male pedipalp 3D PDF (S4 Fig), and G–H were selected from the pushing leg PDF (S5 Fig), as these are considered to be the most anatomically informative. The muscles are depicted in anterior and posterior views to accurately display the main features and shapes, as well as positions relative to neighbouring muscles. The dotted line in C outlines the relative position of the coxal segment. The dotted lines in H outline the trochanter and femur. All scale bars 10 mm.

Muscle 67—This muscle has an origin on the proximal anterior and posterior margins of the coxa and an insertion on the dorsal margin of the trochanter (Fig 5A, 5D and 5E) [27]. The muscle is located between muscles 68 and 69. This muscle has been named the *flexor trochanteris* [52] or the *trochanter levator* [30].

**Fig 6:**
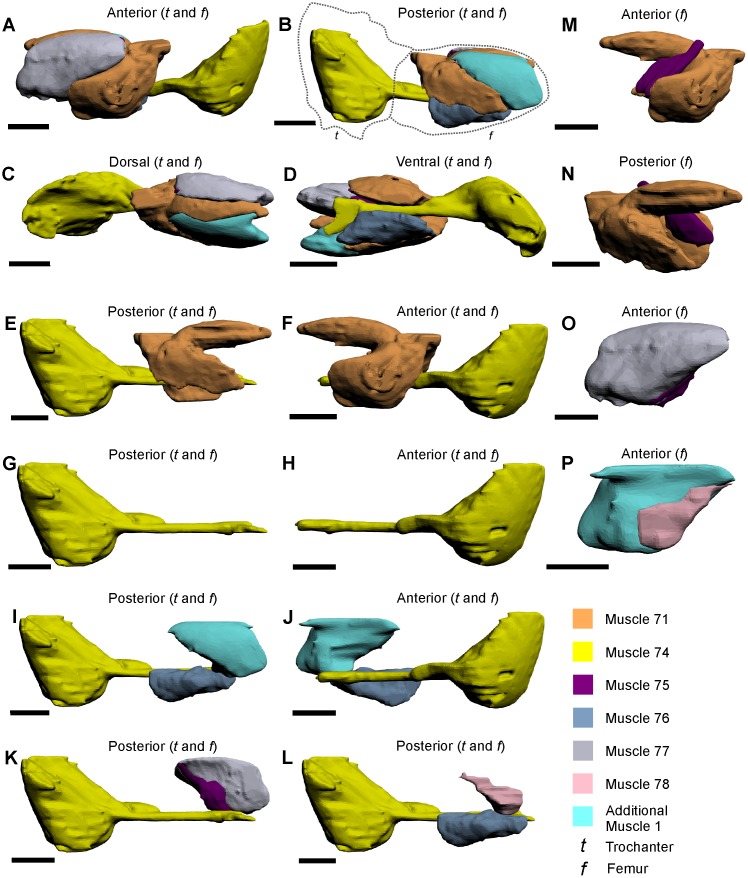
The muscles within the trochanter and femur of the walking legs, male pedipalp and pushing leg, as modelled from iodine staining and micro-CT–scanning. The illustrated muscle reconstructions were selected from the male pedipalp 3D PDF (S4 Fig), as these reconstructions are the most anatomically informative. A–D depict the seven femoral muscles and their relative positions to each other. The individual muscles (E–P) are shown in anterior and posterior views to accurately display the main features, shapes and, where possible, their position relative to adjacent muscles. The dotted lines in B outline the trochanter and femur. All scale bars 5 mm.

Muscle 68—This muscle has an origin on the ventral anterior section of the coxa and an insertion on the anteroventral margin of the trochanter (Fig 5D–5F) [27]. The muscle is located anteriorly relative to muscle 68. This muscle has been named the *trochanter depressor* [30], but is amended here as the *anterior trochanter depressor*.
Muscle 69—This muscle has an origin on the dorsal posterior and anterior sections of the coxa and an insertion on the ventral margin of the trochanter (Fig 5A–5C) [27]. The muscle is located between muscles 67 and 70. This muscle has been named the *flexor basis* [53] or the *trochanter depressor* [30], but is amended here as the *posterior trochanter depressor*.
Muscle 70—This muscle has the origin on the ventral posterior section of the coxa and an insertion on the posteroventral margin of the trochanter (Fig 5A–5C) [27]. The muscle is located posteriorly relative to muscle 69. This muscle has been named the *extensor basis maxillae* [53].
Muscle 71—This muscle has the origin on the dorsal section of trochanter-femur joint and insertions along the proximal anterior, posterior and dorsal sections of the femur (Fig 6A–6F, 6M and 6N) [27]. The muscle is narrow along the dorsal section of the femur and bifurcates into two lobes that flank the anterior and posterior sections of the femur. The muscle is located dorsally relative to muscles 73 and 74, posteriorly relative to muscles 75 and 77, and anteriorly relative to ‘Additional Muscle 1’ and muscle 78. This muscle has been named the *femur levator* [30], *flexor merioni enemiique* [53] or the *ischiopodite extensor* [54].

**Fig 7:**
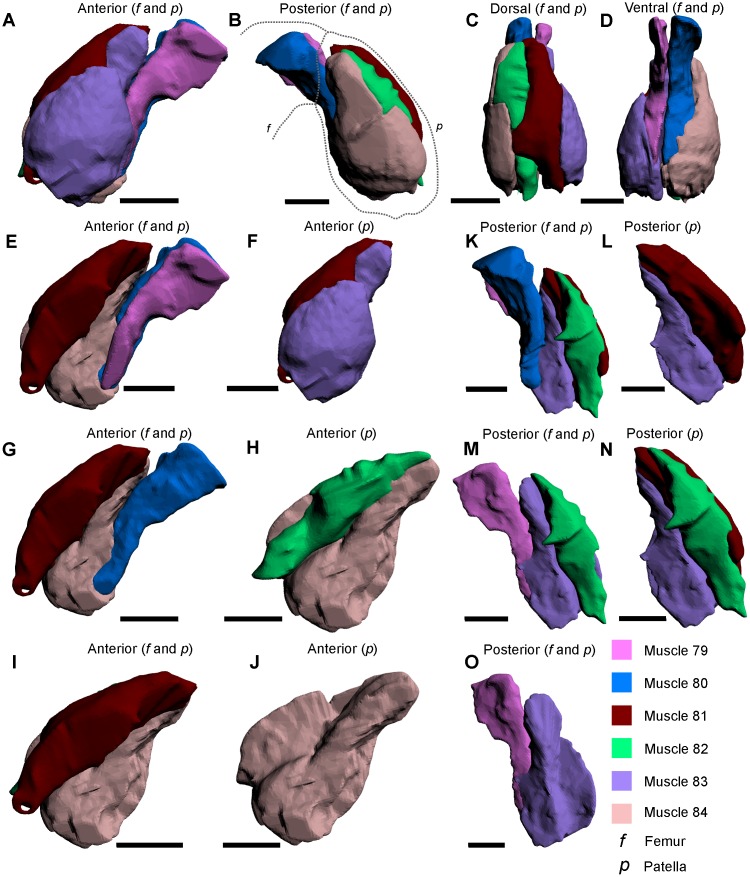
The muscles within the walking legs and male pedipalp patella, as modelled from iodine staining and micro-CT–scanning. The illustrated muscle reconstructions were selected from the male pedipalp 3D PDF (S4 Fig), as these reconstructions are the most anatomically informative. A–D depict the six muscles in four views to illustrate the overall form of the muscles and their relative positions to each other. The muscles (E–O) are shown in anterior and posterior views to accurately display the main features and shapes thereof and, where possible, their position relative to adjacent muscles. The dotted lines in B outline the distal end of the femur and the patella. All scale bars 5 mm.HHh.

Muscle 72—This muscle has an origin on the posterior and ventroposterior sections of the trochanter and an insertion on the posterior ventral margin of the femur (Fig 5G and 5H) [27]. The muscle is located dorsally relative to muscle 74 and proximally relative to muscle 73. This muscle has been named the *femur depressor* [30] or the *ischiopodite flexor* [54].
Muscle 73—This muscle has an origin on the distal anterior section of the trochanter and an insertion on the proximal posterior section of the femur (Fig 5G and 5H) [27]. This muscle is located distally relative to muscle 72. Fourtner & Sherman [54] suggested that this muscle was part of muscle 72 and was referred to as the *ischiopodite flexor*. As muscles 72 and 73 are distinct muscles, this name must be changed, but it is worth noting that the muscle functions as a flexor.
Muscle 74—This muscle has an origin on the anterior and ventral sections of the trochanter and has an insertion on the proximal end of the patellar sclerite (modelled together as one object) (Figs 5G, 5H and 6A–6L) [27, 47]. This muscle attaches to the so-called patellar sclerite, a tendon that that runs the length of the femur [27, 33, 47]. As muscle 74 and the patellar sclerite are grouped together here, muscle 74 is located ventrally relative to muscles 71, 76–80 and ‘Additional Muscle 1’. This muscle has been named the *flexor femoris* [32], *femoro-patella flexor* [30], *merional entapophysis* [53], *mero-carpopodite flexor* [54] or Pringle’s ‘*special muscle*’ [52].
Muscle 75—This muscle has an origin on the middle dorsoanterior section of the femur and an insertion on the distal section of the patellar sclerite of muscle 74 (Fig 6D, 6K, 6M and 6N) [27]. This muscle is very thin, elongate, and is located posteriorly relative to muscle 77 and anteriorly relative to the patellar sclerite. Due to the thin nature of muscle 75, this muscle could not be reliably identified in the micro-CT scan of the walking leg, but is illustrated in the pushing leg and the male pedipalp PDFs. Due to the difficulty in identifying this muscle, it is possible that muscle 75 may be a part of the larger muscle 77.
Muscle 76—This muscle has an origin on the middle ventroposterior section of the femur and has an insertion on the distal shaft of the patellar sclerite (Fig 6B, 6D, 6I, 6J and 6L) [27]. This muscle is located posteriorly relative to the distal half of the patellar sclerite. The muscle has been previously grouped with muscle 74, 77 and ‘Additional Muscle 1’, and called the *mero-carpopodite flexor* [54]. As muscle 76 is a distinct muscle, the name is redundant.
Muscle 77—This muscle has an origin on the distal anterior section of the femur with an insertion on the distal anterior side of the patellar sclerite (Fig 6C, 6D, 6F and 6O) [27]. The muscle runs parallel to the distal anterior margin of the femur. This muscle has been called *femoro-patella flexor* [30], but is amended here to *anterior femoro-patella flexor*. Muscle 77 has been grouped with muscle 74, 76 and ‘Additional Muscle 1’, and called the *mero-carpopodite flexor* [54]. As muscle 77 is a distinct muscle, the name is redundant.
Additional Muscle 1—This muscle has an origin on the distal posterior section of the femur with an insertion on the distal posterior section of patella sclerite (Fig 6B–6D, 6I, 6J and 6P). The muscle is parallel to the distal posterior margin of the femur and located posteriorly relative to muscle 78. This muscle was not illustrated or noted in Shultz’s review, but has been mentioned previously [30, 54]. This muscle has been called the *femoro-patella flexor* [30], but is amended here to *posterior femoro-patella flexor*. ‘Additional Muscle 1’ has previously been grouped with muscle 74, 76 and 77, and called the *mero-carpopodite flexor* [54]. As ‘Additional Muscle 1’ is a distinct muscle, the name *mero-carpopodite flexor* is redundant.
Muscle 78—This muscle has an origin on the distal posterior section of the femur and has an insertion on the distal posterior section of the patellar sclerite (Fig 6L and 6P) [27]. This muscle is very thin and runs the distal length of the posterior side of the patellar sclerite. Due to the thin nature of muscle 78, this muscle was not reliably identified in the walking leg scan, but is illustrated in the pushing leg and the male pedipalp 3D PDFs. Due to the difficulty in identifying the muscle, muscle 77 may be a part of the larger ‘Additional Muscle 1’.
Muscle 79—This muscle has origins on the distal part of the dorsal section of the femur and the anterior and anteroventral sections of the patella, with an insertion on the ventral margin of the tibiotarsus in the walking legs and male pedipalp, and the tibia in pushing legs (Fig 7A–7E, 7K, 7M and 7O) [27]. This muscle is elongate and is located posteriorly relative to muscle 81 and runs parallel with muscle 80. This muscle has been called the *propodite flexor* [54], and the *large leg muscle* [52], but is amened here as the *anterior propodite flexor*.

**Fig 8:**
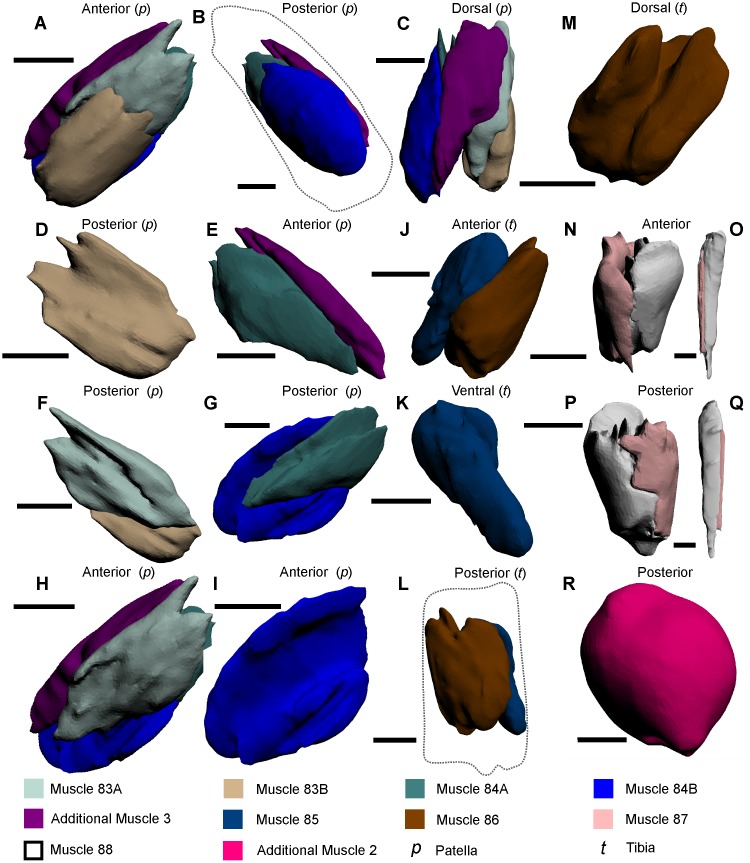
The muscles within the patella, tibia and pretarsus of the pushing leg, and the walking leg and male pedipalp tibiotarsus muscles, as modelled from iodine staining and micro-CT–scanning. (A–C) The five muscles within the patella of the pushing leg in three views to illustrate the overall form of the muscles. (D–I) Each of the five muscles in anterior and posterior views to accurately display the main features and shapes thereof and, where possible, their position relative to adjacent muscles. (J–M) The two muscles within the tibia of the pushing leg. (N, P) The two muscles within the tibiotarsus of the walking leg. (O, Q) The two muscles within the pretarsus of the pushing leg. (R) The newly described muscle within the tibiotarsus of the male pedipalp; due to the subspherical nature thereof, the anterior perspective was not included. Muscle reconstructions A–M, O and P were selected from the pushing leg 3D PDF (S5 Fig). The muscle reconstructions of N and P were selected from the walking leg 3D PDF (S3 Fig). The muscle reconstruction of R was selected from the male pedipalp (S4 Fig). The dotted line in B outlines the pushing leg patella. The dotted line in L outlines the tibia. Scale bars 5 mm for A–N, R; 4 mm for P; 3 mm for O, Q.

Muscle 80—This muscle has an origin on the distal part of the dorsal section of the femur, and the posterior and posteroventral sections of the patella, with an insertion on the ventral margin of the tibiotarsus in the walking legs and male pedipalp, and the tibia in the pushing legs (Fig 7A–7E, 7G and 7K) [27]. This muscle is slender and is located posteriorly relative to muscle 82 and runs parallel with muscle 79. This muscle has been called the *propodite flexor* [54], but is amended here as the *posterior propodite flexor*.
Muscle 81—This muscle has an origin on the anterodorsal section of the patella and an insertion on the anterior process of the tibiotarsus (Fig 7A–7C, 7E–7G, 7I, 7K, 7L and 7N) [27]. The muscle tapers rapidly into an elongated strip that runs through to the anterior process and is located anteriorly relative to muscle 79 and posteriorly relative to muscle 83. This muscle has been called the *propodite extensor* [52], but is amended here as the *anterior propodite extensor*.
Muscle 82—This muscle has an origin on the posterodorsal section of the patella and an insertion on the posterior process of the tibiotarsus (Fig 7B, 7C, 7H, 7K, 7M and 7N) [27]. The muscle tapers rapidly into an elongated strip that runs through to the posterior process. The muscle is located anteriorly relative to muscle 84, posteriorly relative to muscle 80, and runs parallel with muscle 81. This muscle has been called the *propodite extensor* [52], but is amended here as the *posterior propodite extensor*.
Muscle 83—This muscle has an origin on the anterior section of the patella and an insertion on the anterior margin of the tibia (Fig 7A, 7C, 7D, 7F and 7K–7O) [27]. This muscle is located anteriorly relative to muscle 81. This muscle has been named the *anterior patella-tibial flexor* [30]. Fourtner & Sherman thought that muscle 83 was divided into two muscles: the *anterior propodite flexor* and *anterior propodite extensor* [54]. As muscle 83 is clearly one muscle (at least within the walking leg and male pedipalp), both names, the *anterior propodite flexor* and *anterior propodite extensor*, should not be used.
Muscle 84—This muscle has an origin on the posterior section of the patella and an insertion on the posterior proximal margin of the tibiotarsus (Fig 7B–7E and 7G–7J) [27]. This muscle is located posteriorly relative to muscle 82. This muscle has been named the *posterior patella-tibial flexor* [30]. Fourtner & Sherman thought that muscle 84 was divided into two muscles: the *posterior propodite flexor* and *posterior propodite extensor* [54]. As muscle 84 is clearly one muscle (at least within the walking leg and male pedipalp), both names, the *posterior propodite flexor* and *posterior propodite extensor*, should not be used.
Muscle 87—This muscle has an origin on the dorsal section of the tibiotarsus and an insertion on the dorsal margin on the dactylopodite (Fig 8N and 8P) [27]. Muscle 87 is located dorsally relative to muscle 88 and has been named the *abductor dactylis* [32] and the *claw opener* [52]. Fourtner & Sherman split this muscle into the *dactylopodite flexor* and *dactylopodite extensor* [54]. As muscle 87 is clearly one muscle, and the divisions presented in [54] do not reflect what was identified in the micro-CT scans, the names *dactylopodite flexor* and *dactylopodite extensor* should not be used.

Muscle 88—This muscle has an origin on the ventral section of the tibiotarsus and an insertion on the ventral margin of the dactylopodite (Fig 8N and 8P) [27]. This muscle is located ventrally relative to muscle 87. Fourtner & Sherman split this muscle into the *dactylopodite flexor* and *dactylopodite extensor* [54]. As muscle 88 is clearly a single muscle, and the divisions presented in [54] do not reflect what was identified in the micro-CT scans, the names *dactylopodite flexor* and *dactylopodite extensor* should not be used.

### Male pedipalp

The male pedipalp is effectively a modification on the anteriormost pair of walking legs (Figs 3E, 3F, 4E and 4F). While the coxa, trochanter, patella and tibia are the same, the tibiotarsus and dactylopodite are modified during ontogeny into a fortified bulbous section (the tibiotarsus) and a thick, curved claw (the dactylopodite) for mating purposes [26, 31]. The claw is often lost during the first mate pairing and is therefore not often observed [42]. Muscles 67–84 in male pedipalps are the same as those in the walking legs, so are not described again here. However, the muscles within the tibiotarsus are different in these appendages. In the bulbous tibiotarsus of the male pedipalp, a mass of dense muscle (called ‘Additional Muscle 2’) is present, whereas the walking leg tibiotarsus possesses muscles 87 and 88. The muscle mass within the trochanter has, to the knowledge of the authors, never been previously documented.

‘Additional Muscle 2’—This muscle has an origin on the proximal margin of the tibiotarsus and an insertion on the dorsal margin of the tibiotarsus (Fig 8R). The muscle is not immediately adjacent to any other muscles, but is distal to the muscles within the patella. Here it is hypothesized that ‘Additional Muscle 2’ is modified during ontogeny into a single muscle mass to reflect the different use of the appendage and fortify the tibiotarsus and claw for grabbing during mating.

### Pushing leg

The largest of the cephalothoracic appendages is the pushing leg, the main function of which is to push the animal forward, but also to assist in burrowing with the aid of the processes (Fig 3C and 3D) [26, 30]. The pushing leg has nine segments (proximally to distally): the coxal endite that bears a spatulate flabellum (also termed the epipodite [55]) and thick gnathobases; trochanter; femur; patella; tibia; an elongated pretarsus partially surrounded by four processes; and a moveable apotele [26, 27, 31, 47, 53]. Muscles 67–82 in the pushing legs are the same as those found in the walking legs and male pedipalp, so are not described again here. However, muscles 85, 86 and ‘Additional Muscle 3’ are unique to the pushing leg and so are described here. Furthermore, subdivisions of muscles 83 and 84 are presented. Finally, as muscles 87 and 88 in the pushing leg are located within the pretarsus, these muscles are re-described.

Muscle 83A—This subdivision of muscle 83 has an origin on the anterior section of the patella and an insertion on the distal margin of muscle 81 (Fig 8A, 8C, 8F and 8H). This muscle is located anteriorly relative to muscles 80, 81, and 84A. As muscle 83 has been named the *anterior patella-tibial flexor* [30], it is suggested that only 83A be called the *anterior patella-tibial flexor* and 83B be given a different name (see below).
Muscle 83B—This subdivision of muscle 83 has an origin on the centroanterior section of the patella and an insertion on the anterior margin on the tibia (Fig 8A, 8C, 8D and 8F). Muscle 83B is located anteriorly relative to muscle 83A. As muscle 83 has been named the *anterior patella-tibial flexor* [30] (discussed above), it is suggested here that 83B should be named the *anteriormost patella-tibial flexor*.
Muscle 84A—This subdivision of muscle 84 has an origin on the posterior section of the patella and an insertion on the distal margin of muscle 80 (Fig 8A–8C, 8E and 8G). This muscle is located posteriorly relative to muscles 80, 81 and 83A. As muscle 84 has been named the *posterior patella-tibial flexor* [30], it is suggested that only 84A be called the *posterior patella-tibial flexor* and 84B be given a different name (see below).
Muscle 84B—This subdivision of muscle 84 has an origin on the centroposterior section of the patella insertion on the posterior proximal margin of the tibia (Fig 8A–8C and 8G–8I). This muscle is located posteriorly relative to muscle 84A. As muscle 84 has been named the *posterior patella-tibial flexor* [30] (discussed above), it is suggested here that 84B be named the *posteriormost patella-tibial flexor*.
Additional Muscle 3—This muscle has not previously been described and does not resemble any other muscles described here (Fig 8A–8C, 8E and 8H) [27, 33, 47]. The muscle has an origin on the proximal dorsal section of the patella and an insertion on the dorsal margin of the tibia. This muscle is located dorsally relative to muscles 79–84A. Fourtner & Sherman [54] included a possible depiction of this muscle, but did not describe the muscle.
Muscle 85—This muscle has an origin on the anterior section of the tibia and has an insertion on the anterior margin of the pretarsus (Fig 8J–8L) [27]. This muscle is located dorsally relative to muscle 86.
Muscle 86—This muscle has an origin on the anterior and posterior sections of the tibia and an insertion on the posterior margin of the pretarsus (Fig 8J, 8L and 8M) [27]. This muscle bifurcates along the ventral margin of the tibia and is located ventrally relative to muscle 85.
Muscle 87—This muscle has an origin on the dorsal section of the pretarsus and an insertion on the dorsal margin of the apotele (Fig 8O and 8Q). This muscle is elongate, very thin and located dorsally relative to muscle 88. This muscle has been named the *abductor dactylis* [32].
Muscle 88—This muscle has an origin on the ventral section of the pretarsus and an insertion on the ventral margin of the apotele (Fig 8O and 8Q). This muscle is elongate, but thicker than the co-occurring muscle 87 and located ventrally relative to muscle 87.

### Chilaria

These small, spine-bearing appendages are the most posterior, heavily modified and unsegmented cephalothoracic appendages that are also considered to be the first of the opisthosomal appendages (Fig 2G and 2H) [40, 47]. The chilaria have no internal musculature and function mostly as a means of preventing food items escaping behind the pushing legs [47].

### Genital operculum

**Fig 9:**
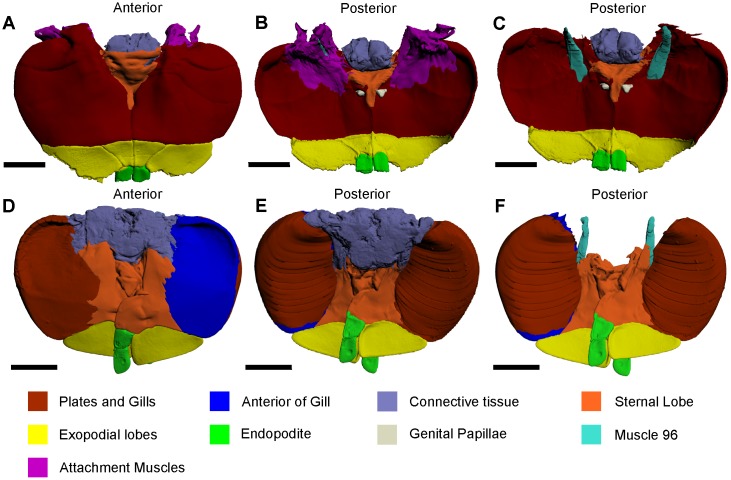
Anterior and posterior views of thoracetronic appendages, as modelled from iodine staining and micro-CT scanning. (A, B) Anterior and posterior views of the genital operculum. (D, E) Anterior and posterior views of a gill operculum. (C, F) Posterior views of the genital operculum and the gill operculum, respectively, with sections removed to depict muscle 96. The reconstructions in A–C were selected from the genital operculum 3D PDF (S7 Fig). The reconstructions in D–F were selected from the gill operculum 3D PDF (S8 Fig). All scale bars 20 mm.HHh.

This opisthosomal appendage is located anteriorly to the gill opercula and attaches to the membrane that connects the cephalothorax and thoracetron (Fig 9A–9C) [47]. The genital operculum covers and protects the other thoracetronic appendages, as well as bearing the sexual organs [42]. The genital operculum consists of two plates, divided by the sternal lobe [27, 31, 39, 42]. Attached to each plate is an exopodial lobe, onto which an endopodite attaches [27, 31, 39, 42]. Additional features include the two genital papillae, the muscle masses that attach the genital operculum to the thoracetron, and the soft tissue that connects the genital operculum to the anteriormost gill operculum. Of the 16 muscles in the genital operculum, only the largest muscle is described and illustrated here, as this was the only muscle that could be definitively identified from the micro-CT scans [27, 47].

Muscle 96 on the genital operculum—This muscle has an origin on the posterior section of the cephalothorax and an insertion on the anterior margin of the genital operculum (Fig 9C) [27]. The muscle is elongate and is located adjacent to the thinner muscle 100, which was not reliably identified in the micro-CT scan and was therefore not figured. This muscle is called the *promotor muscle* [45, 47].

### Gill operculum

The five most posterior appendage pairs of the exoskeleton are the gill (or branchial) opercula that attach to the ventral side of the thoracetron (Fig 9D–9F) [38, 40]. The gill opercula are used for respiration and osmoregulation, and also act as paddles to assist propulsion during swimming [26, 38, 56]. Each pair of gills consists of closely spaced brachial lamellae, of which there are at least 80 in adult individuals [47]. The gill pairs attach to plates that are divided by a sternal lobe [31]. Each plate attaches to an exopodial lobe, while two endopodites attach to the distal sections of the sternal lobe [26, 27, 31, 38, 39, 56]. An additional feature includes the connective tissue that attaches the gill opercula to the thoracetron, but also join the set of thoracetronic appendages [27]. The largest of 16 muscles in each gill operculum is described and illustrated here, as this was the only muscle that could be definitively identified in the micro-CT scans [27, 47].

Muscle 96 on the gill opercula—This muscle has an origin on the thoracetron, at the entapophyseal pits, and an insertion on the anterior margin of the gill opercula (Fig 9F) [27]. The muscle is elongate and located adjacent to the thinner muscle 100, which was not reliably identified in the micro-CT scan and was therefore not figured. This muscle is called the *promotor muscle* [47].

## Discussion

### Muscles in 3D

Studying the muscles of *Limulus polyphemus* using a 3D approach allows researchers interested in chelicerate appendage musculature to consider such anatomy in a biologically accurate manner. The segmentation of muscles identified two new muscles that are described here. This result illustrates how useful *in vivo* muscle studies after iodine staining can be: even with the most careful dissection, muscles can be missed or lumped together unintentionally. Furthermore, digital dissections can be conducted multiple times and one is not constrained by the possibility of the muscles decaying, like standard dissections. The 3D PDFs constructing this atlas offer a resource for researchers interested in visualizing *L*. *polyphemus* muscles, but also general external morphology, in a manner that reflects the three dimensional nature of the organism. Beyond this general interest, there are other important applications of this study. As *L*. *polyphemus* is employed as an outgroup for arachnid phylogenies that include both external exoskeleton and muscle characters [33], 3D reconstructions can aid in more accurate coding. Furthermore, as *L*. *polyphemus* is used as a modern analogue for suggesting the mode of life for extinct taxa, such as the Cambrian arthropod *Sidneyia inexpectans* (Walcott) from the Burgess Shale of Canada [57], and the Silurian eurypterid *Eurypterus tetragonophthalmus* Fischer from Estonia [58], appendage muscle data are useful. An accurate record of *L*. *polyphemus* muscles can help researchers to suggest possible muscle combinations and arrangements within extinct taxa and, by extension, place functional limits on appendage motion and feeding ability.

### Iodine staining

Iodine staining of the appendages to highlight muscles within the exoskeletal segments was an excellent method for studying these anatomical structures in detail. A standard micro-CT scan cannot easily differentiate soft tissue unless the tissue has differing x-ray attenuation properties compared to the surrounding material [59]. As non-biomineralized material has low X-ray absorption and contrast, the addition of iodine solution to the tissue results in the ability to observe soft tissue features in much greater detail [28, 29]. Although the micro-CT scanner would have identified the exoskeleton regardless, the utilization of iodine staining facilitated the clear differentiation of muscles that would not have been possible with unstained specimens. Metscher [28] illustrated this same result with insects, but the present study is the first to extend iodine staining to chelicerates. As a topic of further investigation, it would be interesting to compare the results of micro-CT scans of stained arthropod appendages (or complete specimens) to those that have combined both micro-CT and MRI scans of non-stained specimens.

Studying the scans of iodine-stained specimens in conjunction with anatomical drawings demonstrates that researchers need not conduct physical dissections to identify muscles. This is advantageous in the study of arthropod anatomy, as removing the exoskeleton to identify and examine muscles is a complicated task that often precludes the consideration of their 3D structure. However, care must be taken, as extended periods of staining using this method will dehydrate specimens severely and affect the identification of muscles. This is depicted here in the reconstructions of the walking leg: the muscles within this scan are shrunk in comparison to the pushing leg and male pedipalp (S3–S5 Figs). It is therefore imperative that when conducting iodine staining using the method outlined here, that the specimens not be housed in alcohol for extensive periods. Expanding on the present study using iodine staining to include detailed investigation of other anatomical components of *L*. *polyphemus* may reveal additional, as-yet undocumented internal anatomy of *L*. *polyphemus*. While a study of this magnitude was beyond the scope of this work, the information derived from subsequent studies would be of particular value in arthropod taxonomy and phylogeny, as internal anatomy is often important for the classification of various groups [7].

### Digital specimens

Biological collections housed in various institutions, especially museums, have academic, cultural and historic value, so it is important that specimens be made available (in one way or another) to researchers interested in studying them, but also the public at large. One way is to provide photos and other forms of illustration (e.g., line drawings) in publications, on websites, and other forms of media. However, conventional 2D illustrations of museum specimens can be somewhat limited in the anatomical information they provide. To gain a better understanding of the 3D nature of specimens, it is often necessary for researchers to visit institutional collections or borrow the material in order to physically examine them, which in either case can be impractical for various reasons relating to rarity, fragility, logistics, cost, or policy. Ideally, morphologically unique or interesting museum specimens, not to mention important type material, should be scanned using an MRI, CT or micro-CT scanner for the purpose of re-descriptions, digital dissections, and the production of morphological datasets. Using such an approach, researchers will have access to fundamental, historical and revised taxonomic data. These datasets remove the need for taxonomic housekeeping—the reconsideration of archaic taxon descriptions and fruitless efforts to locate specimens that sometimes no longer exist [60–62]. This is not to suggest that digital records should replace physical specimens, but simply provide researchers with another medium through which organisms can be studied. To see this suggestion become reality, museums and other collection-focused research institutions need to take the lead in compiling the digital collections of useful specimens [25, 28]. This study is an example of how this can be done: an informative museum specimen of *Limulus polyphemus* was used to construct an atlas and re-describe various anatomical features of this important taxon [61].

### Nomenclature

As this atlas re-describes various anatomical parts of *Limulus polyphemus*, a short comment on the general lack of consistency in terminology within the existing literature is warranted. In reviewing the terms assigned to the external exoskeletal features of *L*. *polyphemus*, it became apparent that multiple names for various components of the exoskeleton have been employed, resulting in confusion. Thus, there is a need to standardize the terminology. It is suggested that the names for exoskeletal components assigned in the 3D PDFs be adopted henceforth. The names suggested will hopefully remove any confusion regarding the terminology of the cephalothoracic appendages, especially the most distal segments. Conversely, after considering the wealth of literature on this extensively studied taxon, there are 11 muscles within the cephalothoracic appendages alone that are described, but remain un-named. Therefore, an opportunity exists for arthropod experts to suggest names for these muscles and preclude the need for a numerical system. Assigning names to muscles is especially important as the numbering system has been employed for *L*. *polyphemus* since Lankester (1881) [31].

## Conclusion

*Limulus polyphemus* is described and digitally dissected for the first time in 3D. A CT scan of a large museum specimen depicts the main exoskeletal features, while micro-CT scans of fresh, iodine-stained appendages have facilitated the digitization of appendage segments and muscles. A redescription of the appendage musculature is provided, including the identification of two new muscles, one in the pushing leg (‘Additional Muscle 3’) and one in the male pedipalp (‘Additional Muscle 2’). In addition, two muscles within the pushing leg (muscles 83 and 84) are further subdivided: muscles 83A, 83B, 84A and 84B. This atlas represents the most biologically accurate 3D representation of *L*. *polyphemus* to date, and with a focus on the appendages and their associated muscles, makes the dataset an invaluable resource for researchers interested in the detailed anatomy of this iconic arthropod. The production of 3D atlases for other iconic and rare taxa will create important digital records that will improve accessibility to anatomical information and supplements the physical examination of specimens for taxonomic or other studies.

## Supporting information

S1 Fig 3D interactive model of the complete *Limulus polyphemus* specimen, as modelled from CT-scanning. (PDF)

S2 Fig 3D interactive model of the *Limulus polyphemus* chelicera, as modelled from iodine staining and micro-CT-scanning. (PDF)

S3 Fig 3D interactive model of the *Limulus polyphemus* walking leg, as modelled from iodine staining and micro-CT-scanning. (PDF)

S4 Fig 3D interactive model of the *Limulus polyphemus* male pedipalp, as modelled from iodine staining and micro-CT-scanning. (PDF)

S5 Fig 3D interactive model of the *Limulus polyphemus* pushing leg, as modelled from iodine staining and micro-CT-scanning. (PDF)

S6 Fig 3D interactive model of the *Limulus polyphemus* chilaria, as modelled from iodine staining and micro-CT-scanning. (PDF)

S7 Fig 3D interactive model of the *Limulus polyphemus* genital operculum, as modelled from iodine staining and micro-CT-scanning. (PDF)

S8 Fig 3D interactive model of the *Limulus polyphemus* gill operculum, as modelled from iodine staining and micro-CT-scanning. (PDF)
