# Supplementary figures and images for: A 3D anatomical atlas of appendage musculature in the chelicerate arthropod Limulus polyphemus
Source: PLoS One. 2018 Feb 14;13(2):e0191400. doi: 10.1371/journal.pone.0191400 (PMC5812571; doi:10.1371/journal.pone.0191400)

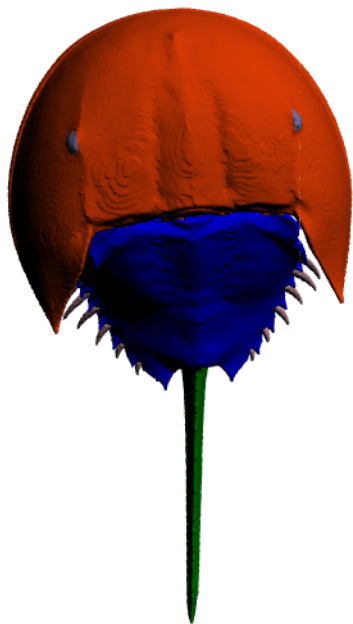

Supplement: S1 Fig — (PDF) [file pone.0191400.s001.pdf]

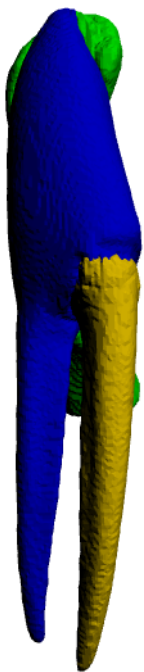

Supplement: S2 Fig — (PDF) [file pone.0191400.s002.pdf]

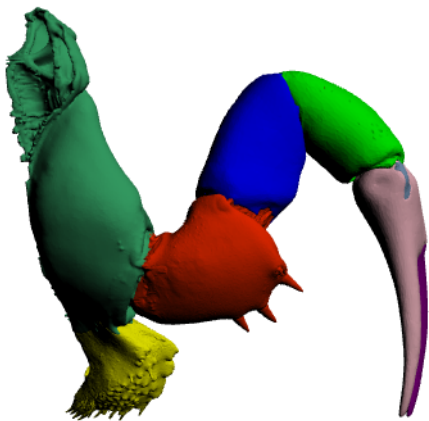

Supplement: S3 Fig — (PDF) [file pone.0191400.s003.pdf]

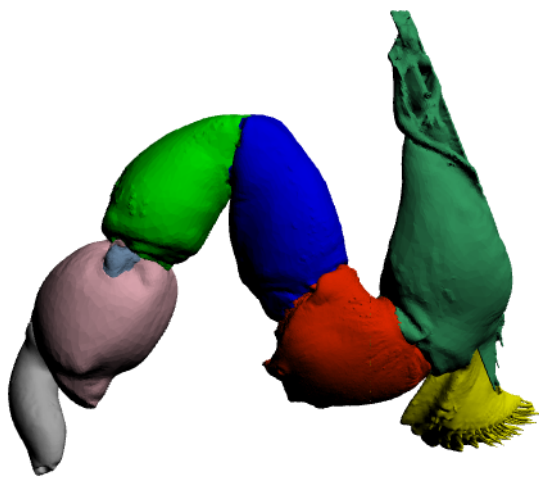

Supplement: S4 Fig — (PDF) [file pone.0191400.s004.pdf]

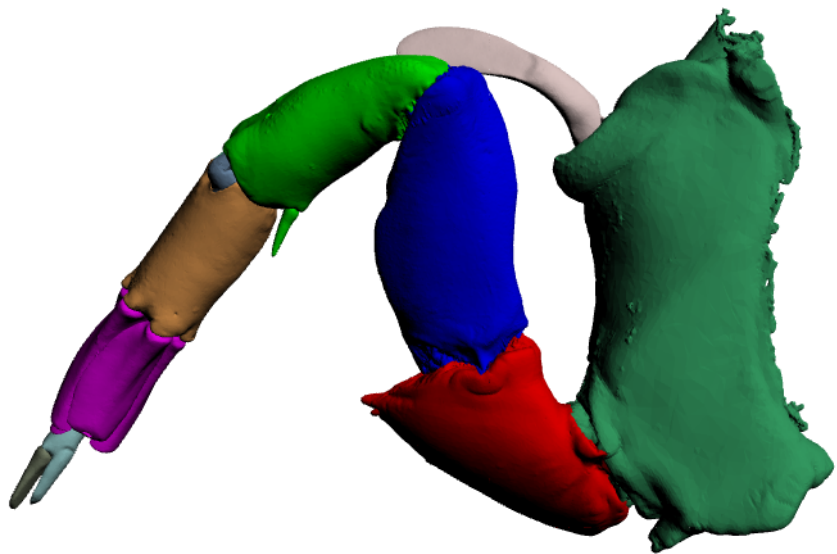

Supplement: S5 Fig — (PDF) [file pone.0191400.s005.pdf]

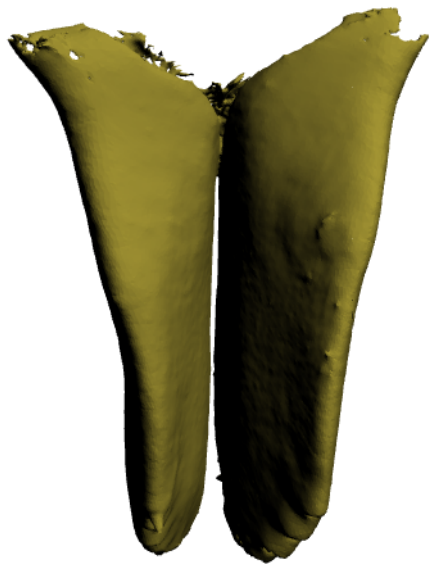

Supplement: S6 Fig — (PDF) [file pone.0191400.s006.pdf]

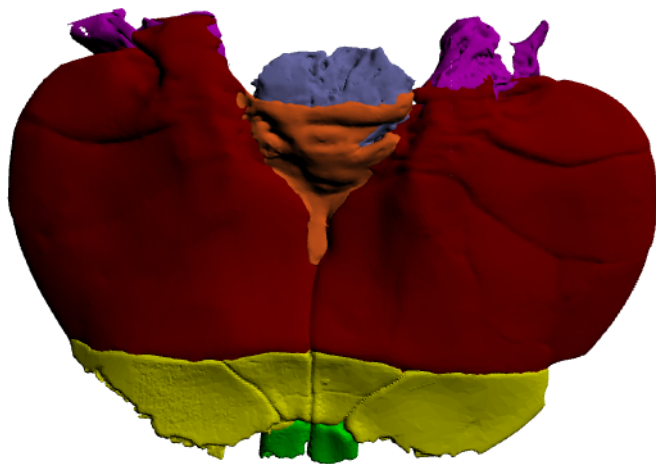

Supplement: S7 Fig — (PDF) [file pone.0191400.s007.pdf]

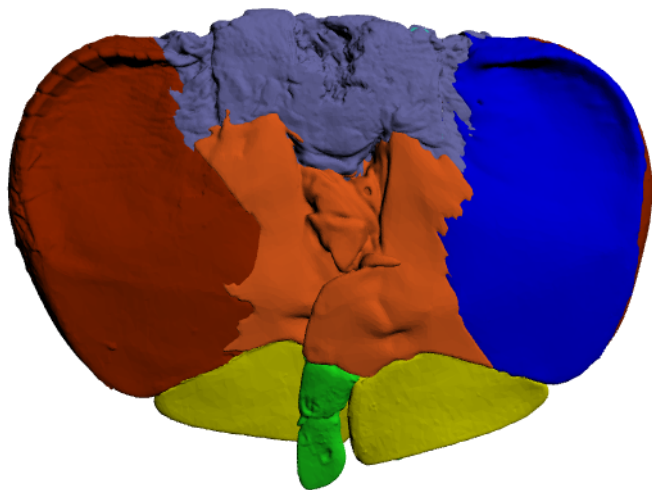

Supplement: S8 Fig — (PDF) [file pone.0191400.s008.pdf]
